# Analysis of cultivated land degradation in southern China: diagnostics, drivers, and restoration solutions

**DOI:** 10.3389/fpls.2025.1533855

**Published:** 2025-02-19

**Authors:** Yanqing Liao, Zhihong Yu, Lihua Kuang, Yefeng Jiang, Chenxi Yu, Weifeng Li, Ming Liu, Xi Guo, Yingcong Ye

**Affiliations:** ^1^ Jiangxi Agricultural University, Jiangxi Province Key Laboratory of Arable Land Improvement and Quality Enhancement, Nanchang, China; ^2^ Technology Innovation Center for Land Spatial Ecological Unprotection and Restoration in Great Lakes Basin, Ministry of Natural Resources (MNR), Nanchang, China; ^3^ Institute of Soil Science, Chinese Academy of Sciences, Nanjing, China

**Keywords:** cultivated land quality degradation, remote sensing indices, SHAP, Yugan County, nature-based solutions

## Abstract

**Introduction:**

Cultivated land quality degradation is a critical challenge to food security, requiring effective nature-based restoration strategies based on comprehensive assessments of land quality. However, existing methods are often costly, limited in scope, and fail to capture the multidimensional complexity of the degradation processes.

**Methods:**

This study integrated vegetation indices, topographic data, and soil physical and chemical properties to construct a model for identifying cultivated land degradation. Remote sensing indices were calculated using Google Earth Engine, enabling large-scale spatial analysis. Machine learning, combined with SHapley Additive exPlanations (SHAP), was employed to explore the driving factors of degradation.

**Results:**

The results indicate that 11.86% of cultivated land in Yugan County is degraded, primarily in the central plain and riparian zones, driven by both natural factors (precipitation, temperature) and anthropogenic factors (straw incorporation, fertilization management). Soil erosion was concentrated in southern hills and near rivers, fertility decline occurred in the central plain, and soil acidification was evenly distributed with generally low degradation levels.

**Discussion:**

Based on these findings, vegetation-based restoration solutions, including deep-rooted crops, crop rotation and intercropping, and straw incorporation, are proposed to address different types of cultivated land quality degradation and support sustainable land management.

## 1 Introduction

The escalating issue of cultivated-land degradation threatens food security and ecosystem stability (Nguyen et al., 2023). According to the Food and Agriculture Organization of the United Nations, approximately one-third of the world’s land faces degradation, primarily due to climate change, over-farming, and irrational land use, leading to soil erosion, nutrient loss, and reduced organic matter, which endanger global food production and ecosystem health (FAO, 2022). If these trends continue, 840 million people will face hunger globally by 2030 and the risk of food supply instability will increase (FAO, 2021). In China, cultivated land quality degradation poses significant threat to national food security, particularly in the southern regions (Fan et al., 2023). Declining soil fertility, nutrient loss, soil erosion, and heavy metal pollution are prevalent issues driven by excessive chemical fertilizer use, monoculture cropping, and intensified soil erosion and water loss (Wang et al., 2020; He et al., 2024). These practices accelerate soil structural damage and reduce biodiversity, further undermining land productivity (Zhang et al., 2021b). As a typical agricultural area in southern China, Poyang Lake exemplifies these challenges, with soil degradation exacerbated by extreme weather events linked to climate change. Problems such as soil erosion, fertility decline, and heavy metal contamination have far-reaching implications for agricultural productivity and ecosystem resilience (Li et al., 2021b). Additionally, urbanization and industrialization intensify resource competition, compounding the degradation of cultivated land (Zheng et al., 2019; Su et al., 2024). Immediate, region-specific protection and restoration measures are essential to mitigate these threats and promote sustainable land management.

Nature-based solutions (NbS) are sustainable approaches that emulate or enhance natural processes to address environmental, social, and economic challenges (Keesstra et al., 2018). NbS emphasize the conservation, restoration, and sustainable management of ecosystems to deliver diverse ecological services, addressing contemporary environmental issues while laying the foundation for future ecological resilience (Cohen-Shacham et al., 2016; Prăvălie et al., 2024). In the context of cultivated land degradation, NbS offer promising strategies for restoring cultivated land quality through vegetation restoration, soil management, and agroecological practices (Sharma et al., 2024). These solutions are vital for mitigating land degradation, enhancing soil fertility, improving water retention, and fostering biodiversity in agricultural settings (Intergovernmental Panel on Climate, C, 2019; Debele et al., 2023). For cultivated land degradation, vegetation restoration not only holds significant importance for ecological rehabilitation in degraded lands but also demonstrates considerable economic benefits, environmental advantages, and operational feasibility (Miralles-Wilhelm, 2021). Moreover, the restoration process enhances biodiversity, improves microclimate conditions, and substantially increases regional ecosystem service provisioning (Sušnik et al., 2022). These benefits are achieved through reduced soil erosion, improved water retention capacity, and enhanced carbon sequestration, providing a critical pathway to achieving ecosystem sustainability (Lal, 2020; Blanco-Canqui, 2024). However, to devise effective restoration strategies for mitigating or reversing cultivated land degradation, the primary challenge is to scientifically identify and assess the types and severity of cultivated land degradation.

Despite its importance, accurately determining the state of land degradation and its driving factors remains a significant challenge. cultivated land degradation exhibits complex spatiotemporal characteristics at a global scale (He et al., 2024). Traditional large-scale, generalized assessments of cultivated land degradation are often costly and fail to support the refined and multidimensional demands of cultivated land management. Incorporating prior knowledge is therefore critical for identifying cultivated land degradation and formulating effective restoration strategies. Vegetation, as an essential indicator of ecosystem health, reflects changes in land conditions (Gaitán et al., 2013). Remote sensing techniques, such as calculating the Ratio Vegetation Index (RVI), Normalized Difference Vegetation Index (NDVI), and Difference Vegetation Index (DVI), have proven effective in identifying areas of soil stress due to their high precision, temporal efficiency, and extensive coverage (Han and Song, 2019). These methods provide a reliable foundation for precise soil sampling and analysis, enabling a deeper understanding of the extent of cultivated land degradation and informing targeted restoration measures (Yan et al., 2021). Furthermore, the complexity of cultivated land degradation processes is exacerbated by the dual influences of climate change and human activities (He et al., 2024). While traditional linear analysis methods can reveal associations between natural conditions, socioeconomic factors, and soil quality, they struggle to capture the nonlinear responses of cultivated land quality to environmental changes (Zhang et al., 2024a). Machine learning (ML) methods address this limitation, yet challenges remain regarding the interpretability of their results (Hussain et al., 2023). SHapley Additive exPlanations (SHAP), a game-theory-based analytical approach, provides a solution by offering a quantifiable and intuitive framework to explain complex ML model outputs (Han et al., 2024). By calculating each feature’s contribution to model predictions, SHAP facilitates the interpretation of model results and enhances transparency in identifying the driving factors behind cultivated land degradation.

Therefore, this study aimed to 1) construct a cultivated land quality degradation diagnosis model for multiple cultivated land quality degradation types by integrating a remote sensing index and the physical and chemical properties of topsoil, enabling quantitative degradation assessment across regions; 2) analyze the cultivated land quality degradation driving factors based on machine learning and SHapley Additive exPlanations (SHAP); and 3) propose vegetation-based cultivated land restoration solutions tailored to different types and levels of cultivated land degradation.

## 2 Materials and methods

### 2.1 Study area

**Figure 1:**
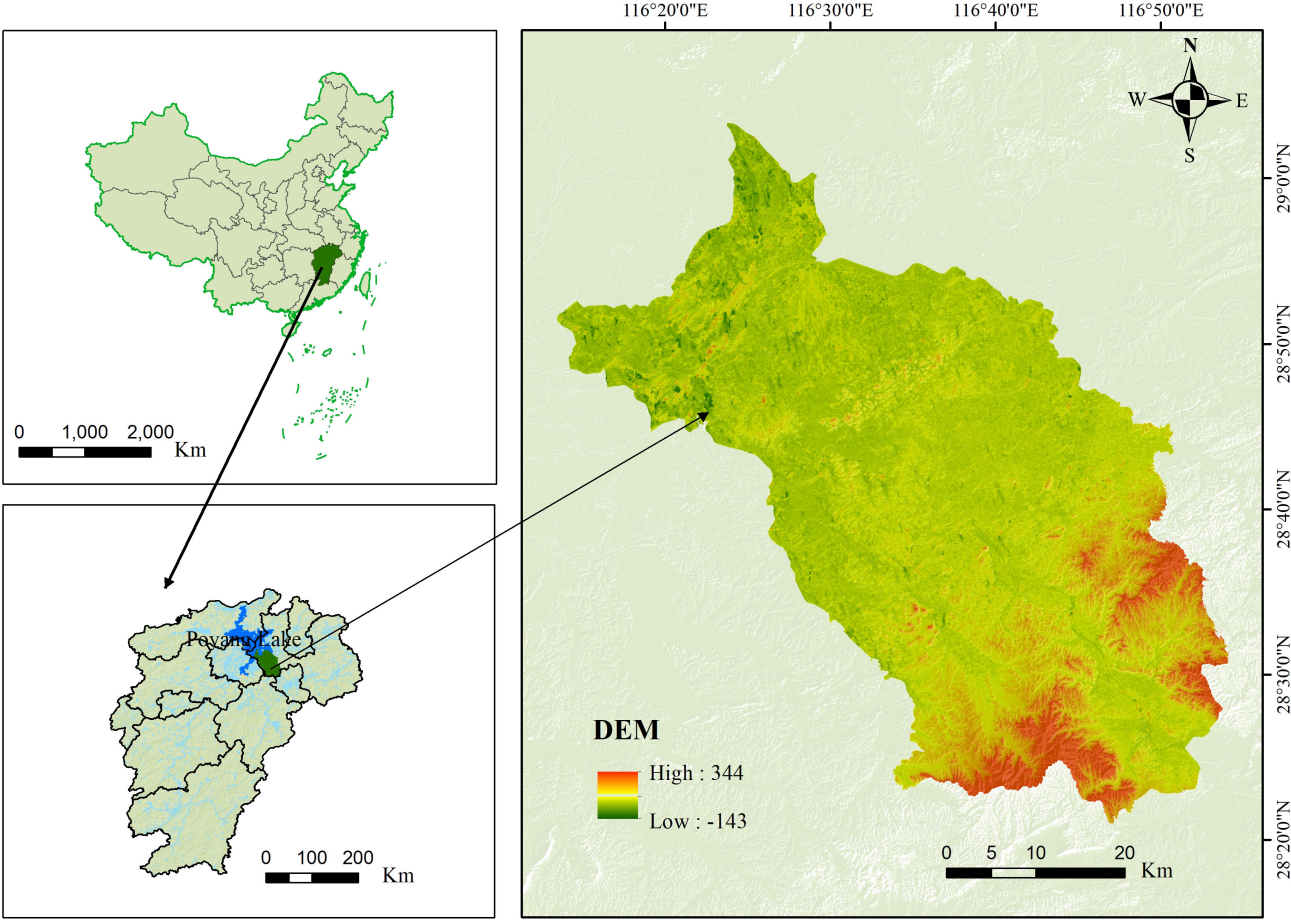
Location of the study area.

**Figure 2:**
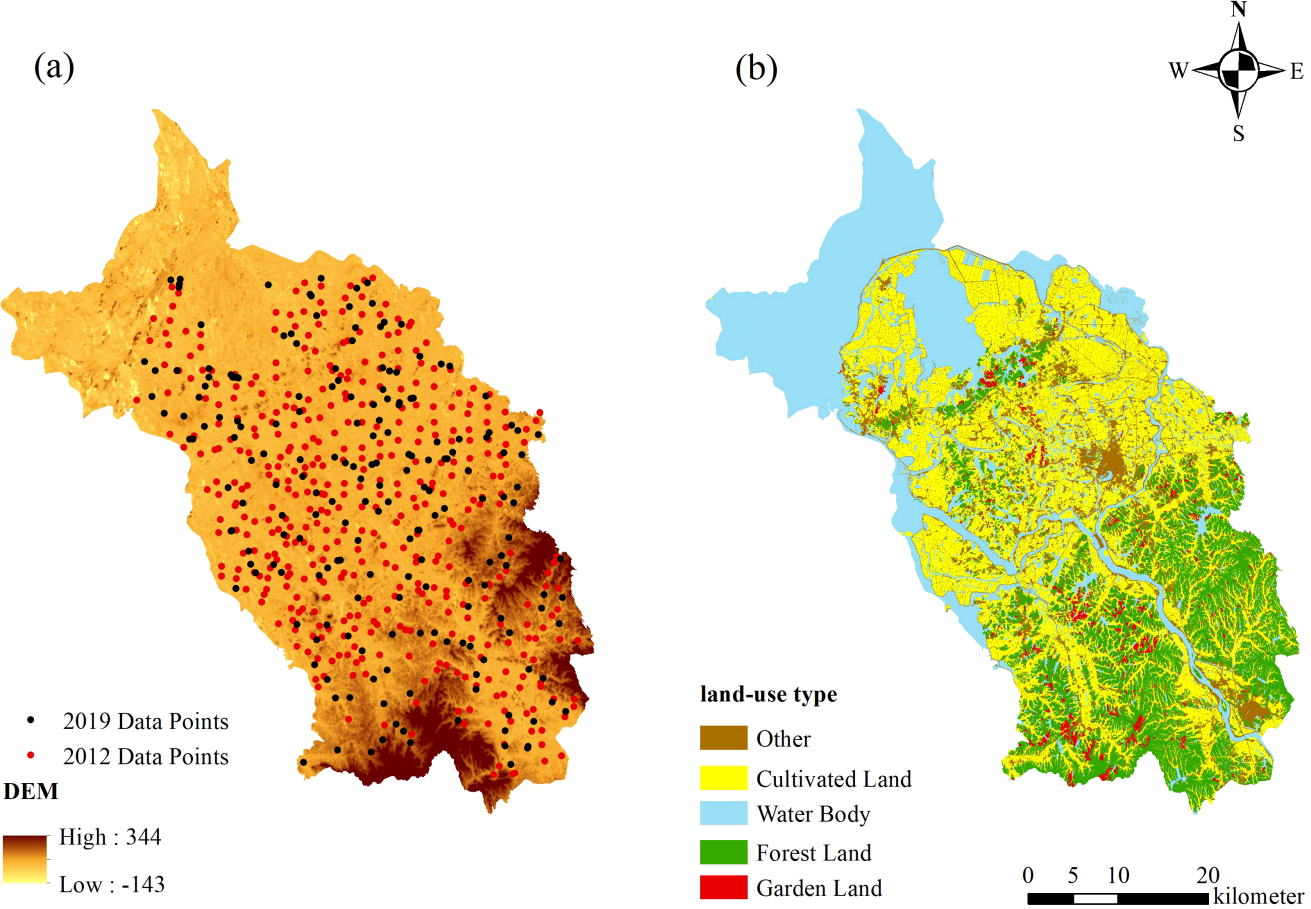
Spatial distribution of sampling sites and land use categories in the study area.

The study area is located in Yugan County, Shangrao City, Jiangxi Province (**Figure 1**). It has a total area of 2,350.36 km^2^ and spans from 28°21’36″ to 29°03’24″ N and 116°13’48″ to 116°54’24″ E (**Figure 1**). It is under the jurisdiction of 9 towns and 11 townships, with a total of 372 village committees. The topography of the city is dominated by low hills and lakeside plains with high terrain in the southeast and low terrain in the northwest. It belongs to a subtropical humid monsoon climate, with an average annual precipitation of 1,548 to 1,692 mm, average annual temperature of 15.4°C to 19.5°C, and frost-free period of 240 to 300 days. Land use mainly consists of arable land and forest land, with arable land accounting for 37.44% of the total area and forest land constituting 19% (**Figure 2**). Rice is the main grain crop, accounting for more than 90% of the total grain production. The soil types are dominated by rice and red soils and vegetation by broad-leaved and coniferous forests. The concentration of cultivated land and gentle slopes in the region make it ideal for studying cropland degradation.

Yugan County represents the typical ecological and agricultural conditions of the Poyang Lake region, characterized by the concentration of cultivated land, gentle slopes, and proximity to the lake’s dynamic water system. Its location within the Poyang Lake basin makes it a hotspot for understanding the interactions between land use, water resource dynamics, and soil degradation. The area is subject to significant land degradation pressures, including soil acidification, soil fertility decline, soil erosion, and soil physical structure degradation, driven by intensive agricultural practices, heavy fertilizer use, and terrain features conducive to runoff and nutrient loss. These issues mirror broader degradation trends across the Poyang Lake region, making Yugan County a critical case for studying the mechanisms and drivers of farmland degradation. Moreover, its role as a major agricultural producer in the region highlights the broader environmental and socio-economic implications of land degradation, such as the risks of reduced crop yields, water eutrophication, and downstream ecological impacts, underscoring its representative significance in regional and national agricultural studies.

### 2.2 Dataset

The data sources covered a wide range of aspects and provided solid support for scientific analysis (**Table 1**).

**Table 1:** Sources of data used in the study.

Typology	Name	Source
Vector data	Soil sample data	Yugan County Soil Testing and Formula fertilization Dataset
	Landmark data	2019 Third National Land Survey Cultivated Land
Remote sensing data	Landsat 7 imagery	Google Earth Engine (GEE) https://developers.google.com/earth-engine/datasets/catalog/landsat-7
	ASTER GDEM V2	Geospatial Data Cloud https://www.gscloud.cn/sources/accessdata
	NASA DEM	EARTH DATA https://earthdata.nasa.gov/esds/competitive-programs/measures/nasadem
Socioeconomic data	Population density	ORNL LandScan Viewer - Oak Ridge National Laboratory: https://landscan.ornl.gov
	Gross domestic product	Registration and Publishing System for Resource and Environmental Science Data (Xu, 2017)
Climate data	Mean annual precipitation	1-km monthly precipitation dataset for China (1901–2023). National Tibetan Plateau/Third Pole Environment Data Center (Peng, 2024)
	Mean annual temperature	China 1-km resolution month-by-month mean temperature dataset (1901–2023). National Tibetan Plateau Data Center (Peng, 2019)
Fertilizer management data	Fertilization data	Yugan County Soil Testing and Formulation Dataset
	Straw return data	Yugan County Soil Testing and Formulation Dataset

The above data in ArcGIS 10.8.1 unified coordinate system.

The topsoil physical and chemical data, including soil organic matter content, pH value, total nitrogen content, and bulk density, along with fertilizer application amount, and straw return amount data were obtained from the Soil Testing and Formula Fertilization Dataset of Yugan County for 2012 and 2019.

Land use data, obtained from the Third National Land Survey of China for 2019 were incorporated to map the spatial distribution of land use types.

Remote sensing data, including Landsat 7 images, were obtained from the Google Earth Engine platform and ASTER GDEM V2 data were provided by the Geospatial Data Cloud (http://www.gscloud.cn/).

Socioeconomic data were obtained from population data from ORNL LandScan Viewer and The China GDP Spatial Distribution Kilometer Grid Dataset - Data from the Registration and Publishing System for Resource and Environmental Science Data (Xu, 2017).

For climate data, 1-km-resolution precipitation and temperature data were provided by the National Tibetan Plateau/Third Pole Environment Data Center (Peng, 2024) and 1-km resolution month-by-month mean temperature dataset (1901–2023) by the National Tibetan Plateau Data Center (Peng, 2019) was used.

### 2.3 Diagnostic model of degree of integrated cultivated land quality degradation

Based on the Cultivated Land Quality Grade Standard (GB/T33469-2016) and relevant documents issued by the Ministry of Agriculture and Rural Affairs, the grading standards for the physicochemical properties of the cultivated land in the study area were determined through comprehensive consideration (**Table 2**). Following the approach proposed by Tang et al. (2023), which emphasizes the integration of natural condition assessments and quality factor thresholds, we adopted a refined method for evaluating cultivated land quality. Specifically, we incorporated their equation for determining quality grades to ensure consistency and accuracy in assessing the degradation thresholds of cultivated land. Cultivated land quality grade was assessed using the 2019 Third National Land Survey Cultivated Land Map as the evaluation unit. The five indicators, including slope, soil organic matter content, soil total nitrogen content, soil pH, and bulk weight, were categorized into four grades based on their respective thresholds. The cultivated land quality grade of each evaluation unit was determined by the cumulative scoring of these indicators in accordance with the method outlined in Equation 1, derived and adapted from the formula used by Tang et al. (2023). This approach allows for a comprehensive evaluation of the degradation and quality status of cultivated land while aligning with region-specific thresholds for sustainable land management.

**Table 2:** Classification criteria for evaluation indicators of arable land.

	Category	First class	Second class	Third class	Fourth class
SOM (g/kg)	Dryland	≥20	15~20	10~15	<10
	Paddy field	≥30	25~30	15~25	<15
TN (g/kg)	Dryland	≥20	15~20	10~15	<10
	Paddy field	≥30	25~30	15~25	<15
pH	Dryland	>=6.0	5.5~6.0	4.5~5.5	<4.5
	Paddy field	>=5.5	5.0~5.5	4.5~5.0	<4.5
BD	Dryland	1.00~1.25	1.25~1.35, 0.90~1.00	1.35~1.45	≥1.45, <0.90
	Paddy field				
SLOPE	Dryland	<=5°	5°~8°	8°~18°	>18°
	Paddy field				

Equation 1 and its detailed parameters are elaborated to align with the methodology proposed in Tang et al. (2023), ensuring a robust framework for cultivated land quality assessment.


(1) $$Grade\{\begin{array}{c} 1,if\;0\leq n_{i2}+n_{i3}\leq 1,n_{i4}=0,\sum_{j=1}^{4}n_{ij}=5\\ 2,if\;2\leq n_{i2}+n_{i3}\leq 3,n_{i4}=0,\sum_{j=1}^{4}n_{ij}=5\\ 3,if\;4\leq n_{i2}+n_{i3}\leq 5,n_{i4}=0,\sum_{j=1}^{4}n_{ij}=5{\;}_{or}\;n_{i4}=1,\sum_{j=1}^{4}n_{ij}=5\\ 4,if\;2\leq n_{i4}\leq 5,\sum_{j=1}^{4}n_{ij}=5 \end{array}$$

where grade is the quality indicator for cultivated land quality evaluation, *n* is the number of indicators, *i* respect the ith evaluation unit, and *j* is the indicator grade. *i* is taken as 1,2,3…n and *j* is taken as 1,2,3,4.

The level of degradation of cultivated land quality was determined using the grade of the two-year cultivated land quality evaluation, calculated using Equation 2.


(2) $$\begin{array}{c} Grade(d)=Grade12-Grade19 \end{array}$$

where Grade(d) is the degradation grade of arable land, Grade12 is the quality grade of arable land in 2012, and Grade19 is the quality grade of arable land in 2019; a positive number represents an increase in grade, that is, no degradation, and a negative number represents a decrease in grade, that is, degradation.

The type of degradation was categorized into four grades: no degradation (the evaluation grade remained unchanged or increased), slight degradation (the grade decreased by one level), moderate degradation (the grade decreased by two levels), and severe degradation (the grade decreased by three levels or more).

### 2.4 Diagnostic model of the degree of single type degradation of cultivated land quality

#### 2.4.1 Soil acidification

Soil acidification refers to the process of soil pH reduction owing to the accumulation of acidic substances, leading to the deterioration of soil physicochemical properties, which in turn affects plant growth and ecosystem functioning (Yadav et al., 2020). The Ratio Vegetation Index (RVI) is a remotely sensed ratio vegetation index that can accurately reflect the degree and spatial distribution of soil degradation (He et al., 2020). The main degradation factors affect soil health and vegetation on the soil surface, leading to changes in RVI. A past study found that RVI reflected soil acidification conditions in southern hilly areas (Bouslihim et al., 2024). Therefore, this study used the GEE cloud-computing platform to perform the band-ratio operation (Equation 3) on remote sensing images and extracted the RVI to indicate the degree of acidification of the soil. RVI was calculated as follows:


(3) $$\begin{array}{c} RVI=\frac{NIR}{RED} \end{array}$$

where *NIR* is the reflectance in the near infrared band and *RED* is the reflectance in the red band.

The change in RVI was significantly correlated with the change in soil pH (P<0.01). We first screened cultivated lands with decreasing RVI and then determined the degree of soil acidification based on the change in pH level.

#### 2.4.2 Soil fertility decline

Soil fertility decline is the process by which the ability of the soil to supply fertilizer and support plant growth declines because of nutrient depletion, particularly total nitrogen, or a reduction in organic matter (Kimetu et al., 2008). Organic matter improves the soil structure, promotes water infiltration and root growth, and provides plants with the necessary nutrients to enhance their water and fertilizer retention capacity and resistance. While nitrogen, as an essential element for plant growth, directly affects nitrogen uptake, utilization, and crop yield (Nguemezi et al., 2020). There was a strong positive correlation between the organic matter and total nitrogen contents(P<0.01); therefore, the organic matter content was chosen as the main soil fertility evaluation index. Normalized Difference Vegetation Index (NDVI) indirectly characterizes soil fertility by reflecting vegetation cover and effectively predicting soil organic carbon content (Zhang et al., 2019). NDVI was calculated as follows:


(4) $$\begin{array}{c} NDVI=\frac{NIR-RED}{NIR+RED} \end{array}$$

NDVI was significantly positively (P<0.01) correlated with soil organic matter and total nitrogen (P<0.01); therefore we chose the area with a declining NDVI tendency and determined the degree of soil fertility decline through changes in soil organic matter content levels.

#### 2.4.3 Soil erosion

Soil erosion refers to the erosion of the top layer of soil caused by rainfall, surface runoff, wind erosion, or irrational land use, which manifests as soil loss, an increase in the sand content of rivers, a decrease in land fertility, and ecosystem degradation (Morgan, 2005). The factors related to soil erosion on cultivated land include slope, water flow, and fertility. Slope is an important factor for measuring the steepness of a terrain, with the greater the slope, the greater the risk of soil erosion (Mei et al., 2024). Soil moisture status is a key indicator for assessing the quality of cultivated land, and the Differential Vegetation Index (DVI) can effectively reflect the regional soil moisture content (Wang et al., 2021). DVI was calculated as follows:


(5) $$\begin{array}{c} DVI=NIR-RED \end{array}$$

There was a significant correlation (P<0.01) between DVI, slope, and organic matter. Based on the soil sampling protocol of this study and previous research findings, we selected cultivated land with slopes greater than 5° that showed a increasing tend in DVI and assessed the degree of soil erosion by examining the changes in soil organic matter content (Mei et al., 2024).

#### 2.4.4 Soil physical structure degradation

Soil physical structure degradation is the process of disruption of the soil aggregate structure, reduction in porosity, and increase in compactness, resulting in the deterioration of water permeability, aeration, and root growth conditions (Hillel, 1998). Bulk density is the mass per unit volume of soil, usually expressed in grams per cubic centimeter or grams per liter (Ferreras et al., 2000). Bulk density reflects the structure and compactness of the soil and has an important influence on soil fertility and aeration. High volumetric weights indicate dense soils with impeded root growth, poor water permeability, and limited gas exchange, all of which affect crop growth (McLenaghen et al., 2017). In contrast, low bulkiness may indicate loose soil with smoother root growth and good water permeability, which favors gas exchange but may also lead to water and nutrient loss (García-Orenes et al., 2005; Omuto, 2008). The higher the soil bulk weight, the lower the soil water content, and the tighter the soil (Fernández-Ugalde et al., 2009). In this study, changes in DVI were significantly negatively correlated with changes in soil bulk density (P<0.01); therefore, these factors were combined to determine the degree of degradation of the soil physical structure. In this study, we selected cultivated land with a DVI declining tendency and then determined the degree of fertility decline via the changes in their soil bulkiness levels.

The types of degradation were categorized into four classes: no degradation (no change or increase in the evaluation rating), slight degradation (one level down), moderate degradation (two levels down), and severe degradation (three levels down).

### 2.5 Random forest model

Random forest (RF) is a widely used integrated learning method for classification and regression tasks, proposed by Breiman and Cutler in 2001. The core idea is to improve the overall accuracy and robustness of the model by constructing multiple mutually independent decision trees and synthesizing the prediction results for each tree (Sheykhmousa et al., 2020; Smith and Wang, 2020). The construction of decision trees by randomly selecting features and data subsets greatly improves the resistance of the model to overfitting and performs well in dealing with high-dimensional data, noisy data, and nonlinear relationships (Jain and Jana, 2023). RF has been widely used in remote sensing image classification and for predicting forest degradation (Gong et al., 2018; Zhou et al., 2023). In this study, the RF model was implemented using the Scikit-learn library for Python 3.11.4. Environmental variables such as topography and climate were selected as inputs, and a spatial distribution map of soil attributes with spatial information was generated by training the model.

The optimal parameters of the model were configured as follows: number of estimation trees (n_estimators) = 300, maximum tree depth (max_depth) = 30, and maximum number of features (max_features) = 1.0. 10-fold cross-validation was used during the model training process and the random seeds of both the training and test datasets were set to 30 to ensure the stability and reproducibility of the results.

### 2.6 Light gradient boosting machine modeling with SHAP

SHAP is a game theory-based analytical method for interpreting the output of complex machine learning models (Huang et al., 2023a). By calculating the contribution of each feature to the model predictions, SHAP provides an intuitive and quantifiable framework for model interpretation. Its core is based on the Shapley value, an assignment method derived from cooperative game theory that measures the impact of each feature on model predictions fairly, thus providing a solid theoretical foundation for the interpretation of complex models (Han et al., 2024). In recent years, SHAP has been widely used in biomedicine, environmental monitoring, and other fields to help decision-makers better understand the importance of features and their impacts on the final results by explaining the prediction mechanism of machine learning models (Zhang et al., 2023a; Du et al., 2024). The contribution rate of each characteristic variable to the target variable, represented by the Shapley value, can be calculated using the following formula.


(6) $$\begin{array}{c} {\text{φ}}_{i}(v)=\frac{1}{|\text{K}|!}\sum_{R}[v(S_{R}^{i}\cup )-v(S_{R}^{i})] \end{array}$$

Where φi represents the Shapley value for feature i, $S_{R}^{i}$ is the subset of features preceding i in a permutation R, ∣K∣ is the total number of features, and $v(S_{R}^{i})$ denotes the predictive contribution of subset $S_{R}^{i}$ ​. The Shapley value quantifies the average marginal contribution of feature iii across all possible feature subsets.

Light Gradient Boosting Machine (LightGBM) is an efficient machine learning algorithm based on gradient boosting designed for processing large-scale data and high-dimensional features (Wei et al., 2021). Its core innovation lies in the leafwise growth strategy, which generates a deeper tree structure and improves the prediction accuracy of the model by prioritizing the leaf nodes with the largest error reduction during expansion. In addition, LightGBM discretizes features using a histogram-based algorithm, which significantly reduces memory occupation and computational cost and improves training efficiency (Wang et al., 2023a). Compared to traditional gradient boosting models, LightGBM can efficiently handle sparse data and large-scale datasets while maintaining good prediction performance and scalability (Lokker et al., 2024).

This study employs LightGBM and SHAP to identify the importance of multiple factors and explore the primary drivers of cultivated land quality degradation. Eleven factors from five aspects of topography, climate, location, socioeconomics, and agricultural management were selected that may affect cultivated land quality degradation, including *Digital Elevation Model (DEM), Slope Direction (ASPECT), Slope Gradient (SLOPE), Mean Annual Precipitation (MAP), Mean Annual Temperature (MAT), Distance to the Rivers (Dist. Rivers), Distance to the Rodes(Dist. Roads), Population Density (POP), Gross Domestic Product (GDP), Straw Return Rate (SRR), Fertilizer Application Rate (FAR)*. This comprehensive selection ensures that various dimensions influencing land quality, such as physical environment, climatic conditions, accessibility, human activity, and agricultural practices, are represented in the model.

The integration of these factors into the LightGBM model, combined with the interpretative power of SHAP, enables a robust analysis of the underlying mechanisms and key contributors to farmland degradation in the study area.

## 3 Results

### 3.1 Analysis of the integrated cultivated land quality degradation

**Figure 3:**
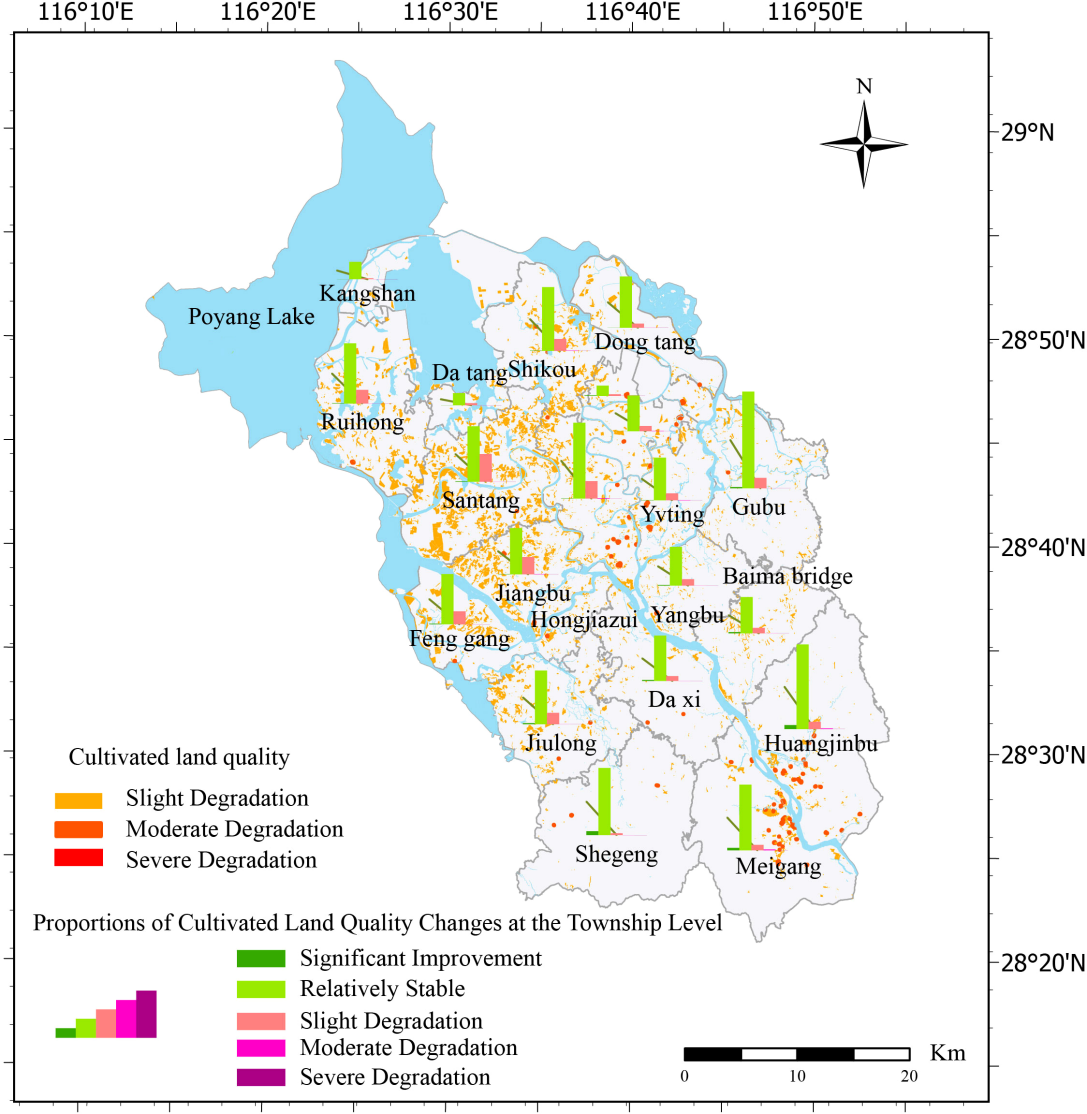
Spatial patterns of integrated cultivated land quality degradation.

Using the cultivated land quality degradation type identification model and degradation degree evaluation method, this study presents a comparative table of the degradation degree of each degradation type in the study area during the decade (**Table 3**). The overall health of cultivated land quality degradation in the study area was good, with only approximately 11.86% showing signs of degradation, of which lightly degraded cultivated land accounted for 11.81% of the total cultivated land, with very little moderate degradation and no heavily degraded cultivated land. To visualize the spatial distribution of the degree of degradation of the overall quality of cultivated land in Yugan County, we measured the degree of degradation in each township and constructed a spatial distribution map (**Figure 3**). Degradation was mainly concentrated in the central and western plains and along the rivers in the southern hilly areas, whereas the degradation degree of the northern lakeshore plain was the least significant.

**Table 3:** Levels of cultivated land quality degradation.

Degradation type		No degradation	Slight degradation	Moderate degradation	Severe degradation	Total degradation
All types	Area (km^2^)	783.0586	104.9531	0.3884	0	105.3416
	Percentage (%)	88.1426	11.8137	0.0437	0	11.8574
soil acidification	Area (km^2^)	853.5768	34.3451	0.4783	0	34.8234
	Percentage (%)	96.0802	3.8659	0.0538	0	3.9198
fertility decline	Area (km^2^)	643.6857	226.9326	17.7661	0.0158	244.7145
	Percentage (%)	72.4545	25.5440	1.9998	0.0018	27.5455
soil erosion	Area (km^2^)	887.2728	0.8959	0.2306	0.0008	1.1274
	Percentage (%)	99.8731	0.1008	0.0260	0.0001	0.1269
Physical structural degradation	Area (km^2^)	888.1472	0.2530	0	0	0.2530
	Percentage (%)	99.9715	0.0285	0	0	0.0285

### 3.2 Analysis of each degradation form of the cultivated land quality

Soil acidification accounted for 3.92% of the total cultivated land area, primarily at the slight degradation level (3.87%), followed by moderate degradation (less than 0.1%) (**Table 3**). Fertility decline made up 27.55% of the total cultivated land quality degradation, predominantly light degradation, which accounted for 25.54% of the total cultivated land area. Moderate degradation represented 2% of the total cultivated land area, significantly higher than other types of degradation, while heavy degradation was observed in only 0.002% of the plots. Soil erosion contributed to 0.03% of total cultivated land quality degradation, mostly slight degradation, with almost no moderate or severe degradation. Lastly, physical structure degradation accounted for 0.13% of the total cultivated land quality degradation, with no cases of moderate or severe degradation. Taken together, most of the degradation types predominantly resulted in slight degradation, especially soil acidification and soil physical structure degradation. However, the proportion of moderate degradation of fertility decline and soil erosion was relatively high, which needs to be studied further. Heavy degradation is rare and occurs only during fertility declines and soil erosion.

**Figure 4:**
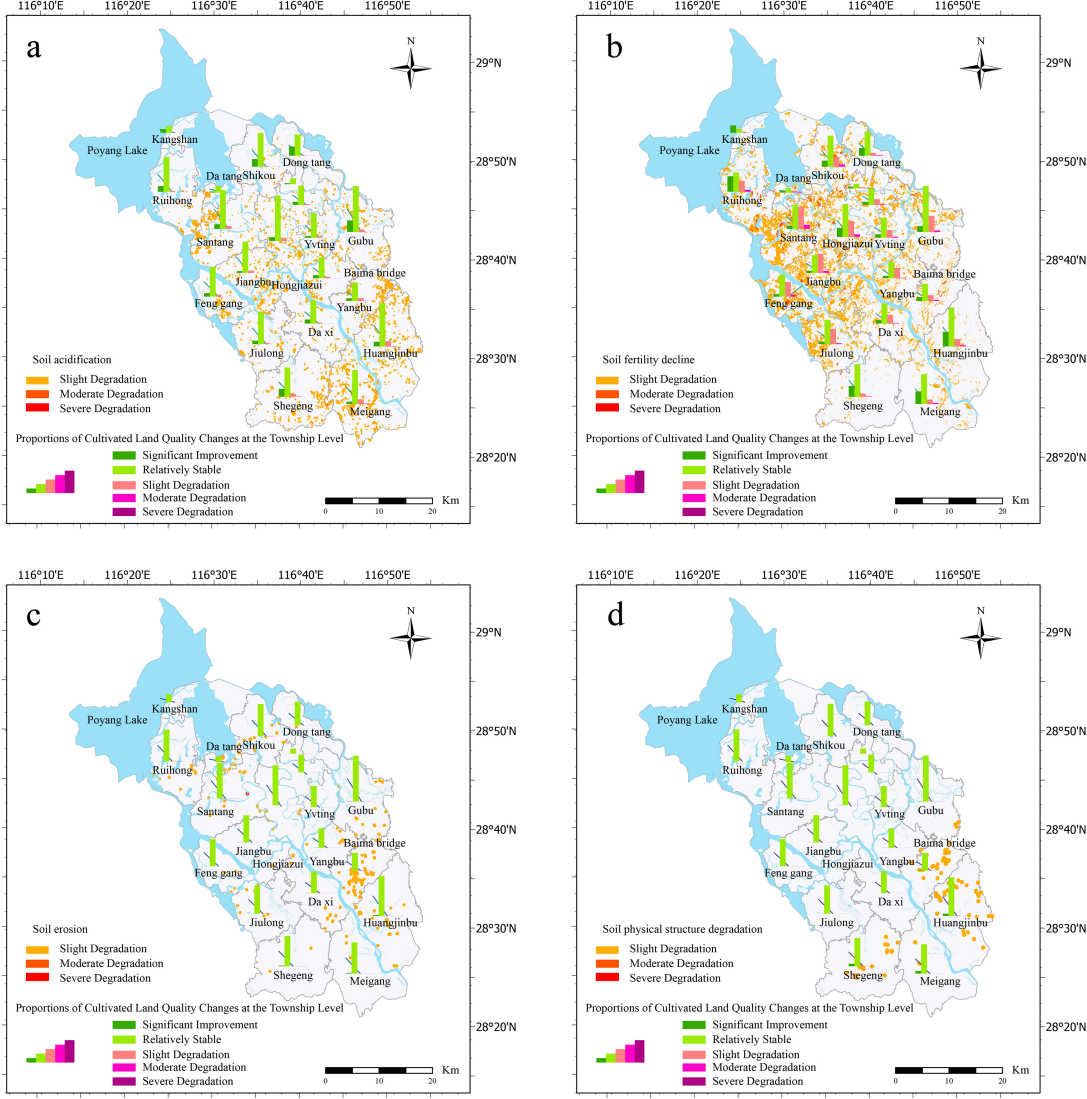
Spatial patterns of degradation levels categorized by degradation types **(A)** soil acidification, **(B)** soil fertility decline, **(C)** soil erosion, and **(D)** soil physical structure degradation.

To visualize the spatial distribution of different types of cultivated land quality degradation and their degree of degradation in Yugan County, we measured the degradation types and degree of degradation in each township and drew corresponding spatial distribution maps (**Figure 4**). The results showed that the spatial differentiation pattern of cultivated land quality degradation presented obvious regional differences. Soil acidification was widely distributed throughout the county, with a lower degree of acidification in the northern lakeshore plain area, whereas acidification is more severe in the central and southern hilly areas, especially in the central area, where acidified cultivated land is more concentrated (**Figure 4A**). In contrast, fertility decline has the widest impact and is particularly problematic in the central, river, and lake littoral areas (**Figure 4B**). Soil erosion was mainly concentrated in the southern hilly areas and along rivers and lakes. Although the overall distribution was small, it had significant impacts in localized areas (**Figure 4C**). In contrast, the area of soil erosion was limited, and the degree of degradation was relatively mild. The physical structure of the soil was degraded to the least extent and was mainly distributed in the southern hilly area, with a relatively scattered and limited range (**Figure 4D**). Comprehensive analysis showed that fertility decline in the central part and soil acidification in the south were the most prominent problems, whereas the impacts of soil erosion and physical structure degradation were more localized.

### 3.3 Drivers influencing the cultivated land quality degradation

**Figure 5:**
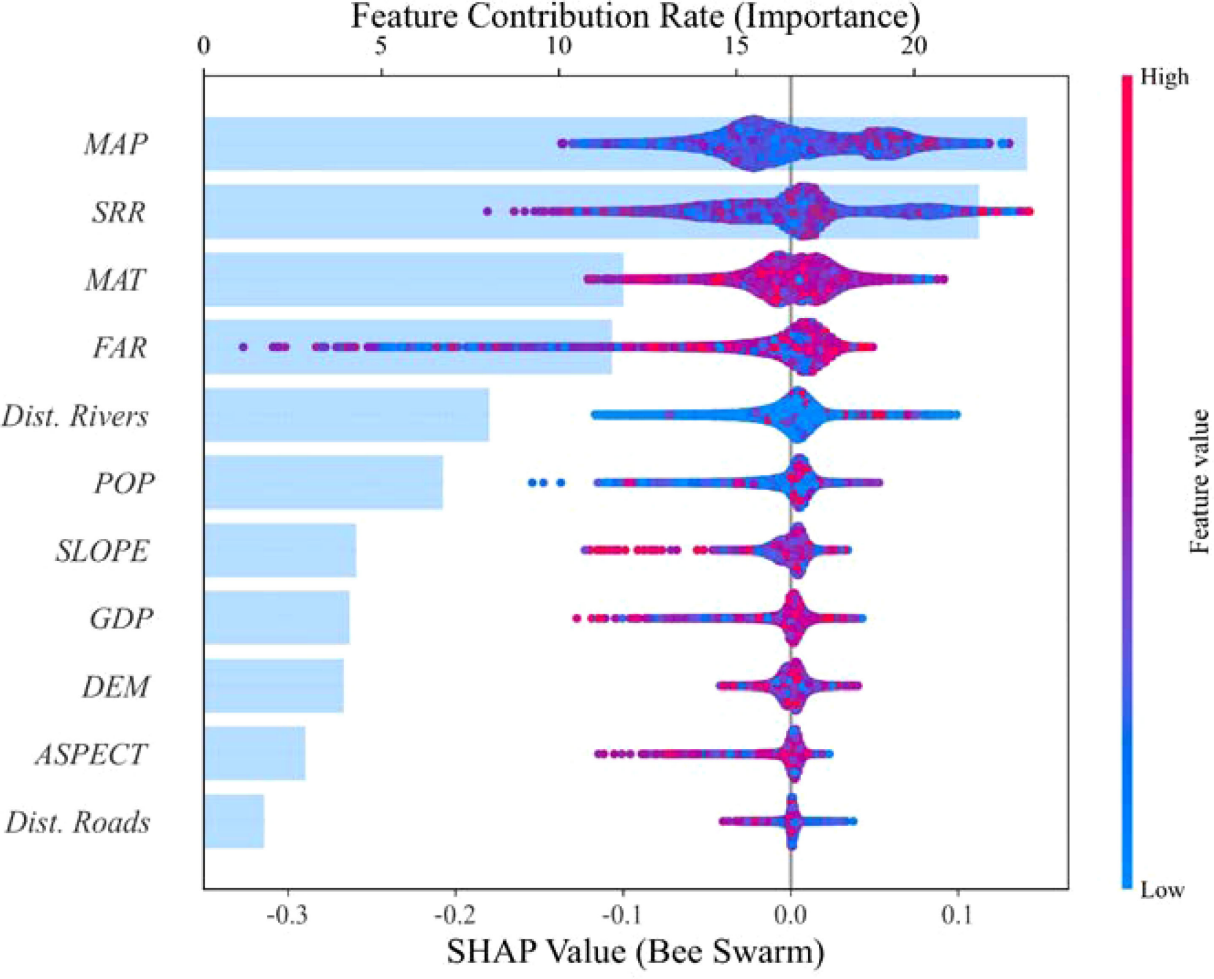
Importance ranking of impact factors based on LGB-SHAP mode.

**Figure 6:**
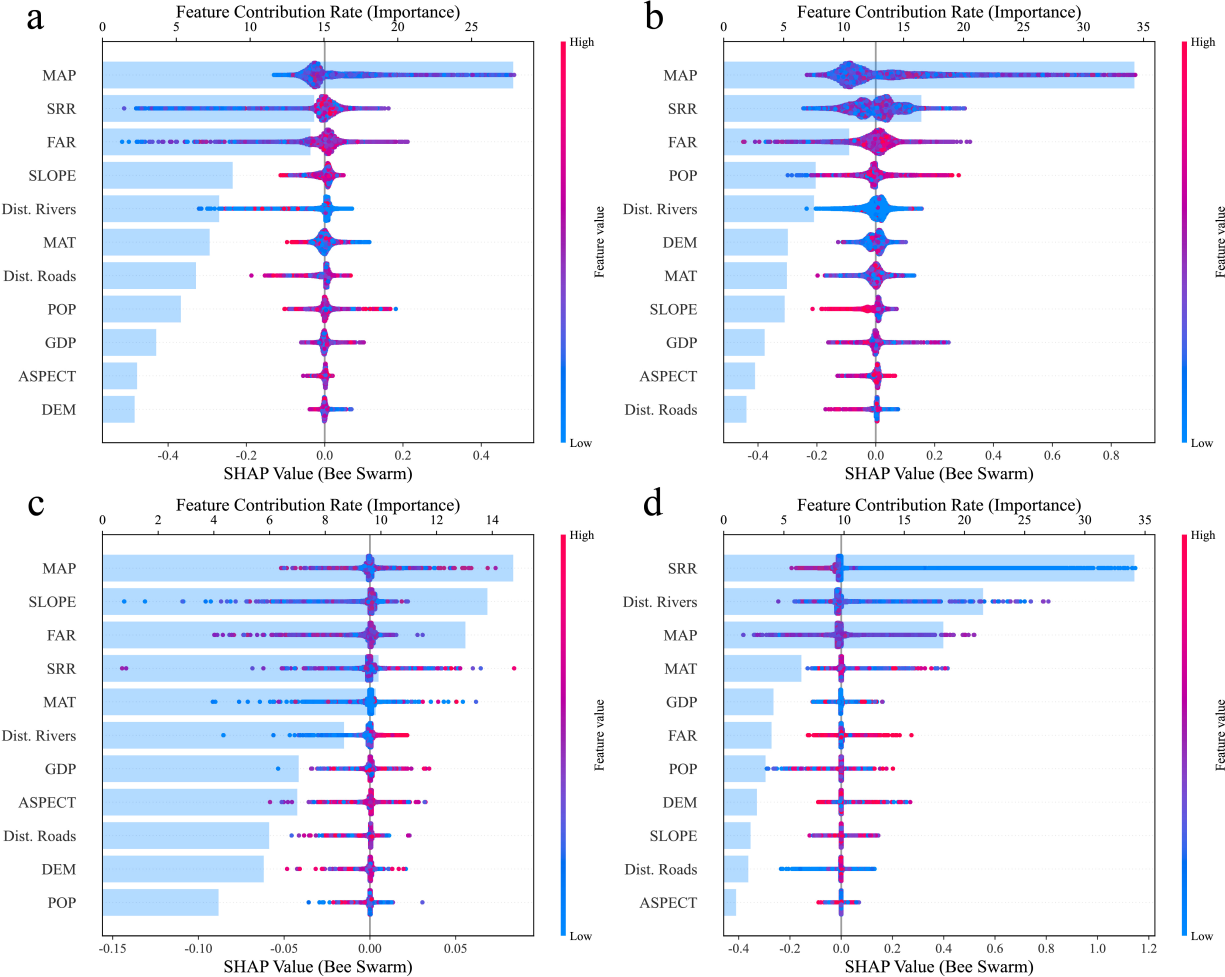
Importance ranking of impact factors for different degradation types based on the LGB-SHAP model **(A)** spatial distribution of soil acidification, **(B)** spatial distribution of soil fertility decline, **(C)** spatial distribution of soil erosion, and **(D)** spatial distribution of soil physical structure degradation.

In this study, 11 factors influencing the degradation of cultivated land quality were systematically selected, encompassing five dimensions: topography, climate, geographic location, socio-economic conditions, and agricultural management practices. After the VIF test (VIF < 5) for the 11 factors, it was confirmed that there was no multicollinearity problem for these independent variables. On this basis, the influence characteristics of each driving variable on the degradation of comprehensive quality of cultivated land and different cultivated land quality degradation types were analyzed by the SHAP value interpretive method, and the results are shown in **Figure 5** and **Figure 6**. The target variable for this study is the score for a particular type of degradation, where an increase in the score indicates mitigation of degradation and vice versa. For example, an increase in the soil acidification score mitigates acidification, whereas a decrease in the score intensifies acidification.

By analyzing the SHAP values, this study revealed the main factors influencing the degradation of each type of cultivated land. Overall, MAP and agricultural management factors had significant effects on the different types of degradation but the drivers of each degradation type differed. For soil acidification, MAP was the most important mitigating factor, and its increase significantly reduced the acidification risk, whereas the excessive application of fertilizers exacerbated the acidification process. The fertility decline was mainly driven by precipitation and straw return rate, both of which increased to help mitigate the fertility decline, whereas excessive fertilizer application exacerbated this problem. Soil erosion was mainly affected by slope, with larger slopes exacerbating erosion; however, appropriate increases in annual precipitation and rational fertilizer use helped to mitigate it. The degradation of the soil physical structure was more mitigated by the straw return rate; however, it tended to be exacerbated in areas with greater slopes and the degradation was more pronounced farther away from the river. Changes in the overall cultivated land quality were also positively correlated with precipitation and straw return rate but excessive fertilizer application negatively affected the overall quality.

## 4 Discussion

### 4.1 Tendency of cultivated land quality degradation

This study indicates that cultivated land in the Poyang Lake area is facing a series of degradation risks, including soil acidification, soil fertility decline, soil erosion and soil physical structure degradation, a finding that is consistent with existing research. For example, Zeng et al. conducted a spatial assessment of farmland soil pollution across China, revealing similar degradation trends in other regions, such as a decline in soil fertility and the accumulation of heavy metals, which may lead to significant ecological and health risks (Zeng et al., 2019). In the Poyang Lake area, the long-term application of chemical fertilizers has led to a decline in soil organic matter content, looser and less stable soil structure, and fertility decline (Liu et al., 2023). Excessive use of nitrogen and phosphorus fertilizers breaks the acid-base balance of the soil, which affects the healthy growth of the crops and increases the risk of soil acidification (Huang et al., 2012). Intensive land use in areas with high precipitation and complex terrain has led to the destruction of vegetation and degradation of the soil physical structure, triggering serious soil erosion and nutrient loss (Yuan et al., 2016; Zhang et al., 2021b). These issues are further corroborated by Zeng et al., who highlighted the widespread challenges of soil fertility decline and contamination across China (Zeng et al., 2019). If these issues are not addressed, they could lead to a series of environmental and socio-economic problems, including water eutrophication (Cai et al., 2017), acid rain, and a decline in crop yields (Zhang et al., 2021a), which would severely impact both the environment and local livelihoods. This study further highlights that the most prominent risk in the region is the degradation of arable land fertility, which poses a significant threat to sustainable agriculture. Given the urgency of this issue, it is critical to implement nature-based solutions (NbS) and other adaptive management strategies to mitigate the negative impacts and restore soil health.

### 4.2 Drivers influencing cultivated land quality degradation

The mean annual precipitation, straw return rate, and fertilizer application rate exhibited important effects on various types of cultivated land quality degradation. High precipitation exacerbates soil erosion, especially on slopes or in areas where soil and water conservation measures have not been implemented. Precipitation in the Poyang Lake Basin is concentrated during the rainy season and its complex topography makes precipitation-induced erosion a serious problem (Chen et al., 2020). Under excessive precipitation, topsoil is easily washed away, leading to the loss of soil organic matter and nutrients, a gradual decline in soil fertility, and destruction of the physical structure of the soil, making it loose and unstable (Zhan et al., 2023). In addition, precipitation affects the acid-base balance of the soil and can exacerbate soil acidification, especially under the condition of long-term application of nitrogen fertilizer (Guo et al., 2018). Higher precipitation increases the risk of nutrient loss, especially soluble nutrients, such as nitrogen and phosphorus, which are more likely to be leached by rainfall into groundwater or discharged into river systems, weakening soil fertility (Siddique et al., 2020). In terms of soil structure, excessive precipitation leads to the dispersion of soil particles, weakening the water-holding and carrying capacity of the soil, making it more susceptible to erosion, and thus accelerating the degradation of the physical structure of the soil (Jin et al., 2021).

Straw returning can increase soil organic matter content, improve soil structure, and alleviate soil acidification (Hu et al., 2023b). Straw returning strengthens the carbon and nitrogen cycles and increases soil microbial activity, thus enhancing the water and fertilizer retention capacity of the soil and reducing the risk of soil erosion (Shi et al., 2022). In addition, straw returning can reduce the dependence on chemical fertilizers and the associated problems stemming from their overuse, including increased soil degradation (Wang et al., 2023b) and acidification and decreased soil fertility (Islam et al., 2023).

Runoff and leaching transport excess fertilizer nutrients into water bodies, aggravating eutrophication and pollution problems (Zhang et al., 2021a). To address overfertilization, fertilizer management strategies such as soil testing and formulation have been developed. Precise fertilization plans can reduce the waste of chemical fertilizers and alleviate soil acidification and water pollution (Smith and Siciliano, 2015). Appropriate farmland management practices, such as the combination of straw return and rotational cropping systems, can also significantly improve soil health and promote the sustainable use of arable land (Zhang et al., 2024b).

This study found that the effect of river distance on the degradation of soil physical structure in the Poyang Lake area was particularly significant, supporting the results of past studies (Yuan et al., 2016; Li et al., 2021a). Simultaneously, population growth had a significant effect on the fertility decline of arable land, which was observed in past studies (Bao et al., 2021; Liu et al., 2021).

### 4.3 NbS measures for addressing the degradation of cultivated land quality

Vegetation-based cultivated land restoration solutions offer ecologically friendly and economically feasible strategies for addressing cultivated land degradation by optimizing vegetation selection and management (Feng et al., 2022). These solutions leverage the ecological functions and regulatory capacities of vegetation to effectively mitigate various types of soil degradation (Seddon et al., 2020). In the Poyang Lake region, such approaches have shown significant potential and effectiveness in tackling key challenges, including soil acidification, soil fertility decline, soil erosion, and soil structural degradation (Zhang et al., 2023b).

Soil acidification, the prominent degradation issue in the Poyang Lake region, often results from prolonged fertilizer use and acid rain. In this context, Cerda et al. (Spain) found that planting acid-tolerant species and employing vegetative cover are essential for mitigating soil acidification. For example, in acidic soils, *Lolium perenne* and *Trifolium repens* improve soil microbial activity and enhance buffering capacity through root exudates (Cerdà et al., 2022). Furthermore, straw incorporation has proven to be a highly effective strategy for neutralizing acidic soils. Research shows that applying 6–10 tons of straw per hectare significantly raises soil pH and increases the availability of calcium and magnesium ions. This method chemically balances soil acidity through the release of organic carbon and carbonate ions during straw decomposition (Chen et al., 2023). Such integrated vegetation management strategies provide a scientific foundation for the sustainable development of agriculture in the region.

Cultivated land fertility decline, largely caused by long-term monocropping and nutrient overexploitation, remains a critical issue. A typical approach is to plant cover crops, such as legumes like *Medicago sativa* and *Trifolium repens*, which, by forming symbiotic relationships with rhizobia, significantly increase soil nitrogen content and improve microbial activity in the soil (Lal, 2022). Furthermore, implementing crop rotation and intercropping systems, such as alternating leguminous crops with cereal crops, effectively alleviates nutrient depletion caused by monoculture practices and promotes nutrient cycling (Zhang et al., 2016). Studies in the Poyang Lake region indicate that incorporating 6–10 tons of straw per hectare, combined with moderate nitrogen fertilization (30–50 kg/ha), can substantially increase soil organic matter and enzyme activity, thereby enhancing nutrient availability. This practice releases significant amounts of nutrients during straw decomposition while optimizing microbial community structure, effectively restoring soil fertility (Lin et al., 2017; Hu et al., 2023a).

Soil erosion presents a major challenge for sloped cultivated lands in the Poyang Lake region, particularly during the rainy season. Vegetative buffer strips, an essential vegetation-based solution, effectively reduce surface runoff and soil erosion. The research conducted in the Poyang Lake region showed that using vegetative buffer strips with species such as *Salix* spp. and *Phragmites australis* along sloped cultivated lands not only reduces sediment loss but also contributes to stabilizing soil structure (Huang et al., 2023b). Similarly, straw mulching has proven highly effective in preventing soil erosion. Studies reveal that applying 6–8 tons of straw per hectare can reduce soil erosion rates by 35%–50% (Xing et al., 2023). In southern China, deep-rooted plants such as *Medicago sativa* significantly improve soil infiltration and stability, reduce erosion and enhance water retention and utilization efficiency (Xie et al., 2022). Additionally, *Robinia pseudoacacia*, used as a biological barrier, provides effective runoff interception and enhances soil stability on sloped lands through its deep-root system and dense canopy (Pan et al., 2024).

Soil physical structural degradation, characterized by compaction, reduced porosity, and poor aeration, significantly impairs root growth and water circulation. In the Poyang Lake region, planting deep-rooted crops like *Brassica napus* and *Beta vulgaris* effectively addresses these issues. The deep roots of these crops penetrate compacted layers, improving aggregate stability and enhancing soil aeration and water retention (Zhang et al., 2014). Additionally, incorporating biochar derived from straw into the soil has emerged as an innovative solution. This practice not only increases soil porosity and water retention but also creates favorable habitats for microorganisms, further enhancing soil ecological functionality (Xu et al., 2021). These solutions provide a scientific pathway for restoring heavily degraded cultivated land in the Poyang Lake region while underscoring the critical role of vegetation in soil health management.

In conclusion, vegetation-based natural solutions fundamentally improve soil health and ecosystem service capacity through scientific vegetation management (Qiu et al., 2024). From employing cover crops to enhance fertility to utilizing deep-rooted plants and straw incorporation technologies to improve soil structure, these strategies highlight the central role of vegetation in mitigating cultivated land degradation. Additionally, straw incorporation, as a circular utilization of vegetative resources, demonstrates comprehensive benefits in enhancing soil fertility, alleviating acidification, and stabilizing soil structure. Moving forward, integrating digital agriculture technologies and region-specific management strategies will be crucial for optimizing these solutions and achieving greater ecological and economic benefits.

### 4.4 Study limitations

While this study advanced the understanding of cultivated land quality degradation risks and drivers in the Poyang Lake area, there were some limitations. First, although the use of LightGBM and SHAP improved the accuracy to a certain extent, these models have limitations in capturing complex nonlinear relationships and may fail to comprehensively reflect all potential influencing factors. In addition, key variables, such as socioeconomic factors, were not fully incorporated into the analytical framework, which may have led to the limited comprehensiveness of some of the findings. Furthermore, the data and indicators used in this study may have some measurement errors, and the accuracy of the soil’s physical and chemical properties may have been affected by differences in local monitoring methods. Therefore, future studies should consider expanding the scope of data collection, incorporating more variables, and exploring different analytical models to comprehensively reveal the complex mechanisms of cultivated land quality degradation.

## 5 Conclusions

This study analyzed cultivated land quality degradation in Yugan County, examining the spatial distribution characteristics of different degradation types and their driving factors using remote sensing, GIS technologies, LightGBM, and SHAP. The results revealed significant spatial differences in cultivated land return degradation, with fertility decline being particularly serious. The mean annual precipitation, straw return rate, and chemical fertilizer application rate were the main driving factors of cultivated land quality degradation. The degree of soil acidification was higher in the central and southern hilly areas and lower in the northern lakeshore plains. Fertility decline was especially obvious in the central area and along the rivers and lakes, soil erosion was mainly concentrated in the southern hilly areas with larger slopes, and soil physical structure degradation was concentrated in the southern hilly areas but with a fragmented distribution and relatively small influence.

This study not only reveals the spatial pattern of cultivated land quality degradation in Yugan County but also provides a scientific foundation for understanding the primary drivers of different types of degradation. These findings offer valuable guidance for the development of regional strategies for cultivated land unprotection and degradation management, particularly in addressing acidification and fertility decline in the central and southern parts of Yugan County to promote the sustainable development of regional agriculture.

## Data Availability

The original contributions presented in the study are included in the article/supplementary material. Further inquiries can be directed to the corresponding author.
